# Current landscape of clinical use of *ex vivo* expanded natural killer cells for cancer therapy

**DOI:** 10.31744/einstein_journal/2024RW0612

**Published:** 2024-11-12

**Authors:** Júlia Teixeira Cottas de Azevedo, Juliana Aparecida Preto de Godoy, Cláudia de Souza, Micheli Severo Sielski, Larissa Leggieri Coa, Augusto Barbosa, Lucila Nassif Kerbauy, Andrea Tiemi Kondo, Oswaldo Keith Okamoto, Nelson Hamerschlak, José Mauro Kutner, Raquel de Melo Alves Paiva

**Affiliations:** 1 Hospital Israelita Albert Einstein São Paulo SP Brazil Hospital Israelita Albert Einstein, São Paulo, SP, Brazil.

**Keywords:** Natural killer cells, Neoplasms, Immunotherapy, Cell- and tissue-based therapy, Cell culture techniques, Feeder cells

## Abstract

Natural Killer cells are immune leukocytes required for responses against tumor cells and virus-infected cells. In the last decade, natural killer cells have emerged as promising tools in cancer therapy, and clinical studies on patients treated with natural killer cells have revealed increased rates of disease-free survival. In this article, we review results from the major clinical trials that have used natural killer cells for cancer treatment, including their global distribution. We also discuss the major mechanisms of natural killer cell activation and expansion and focus on the advantages and disadvantages of each mechanism for clinical applications. Although natural killer cells can be isolated from several sources, primary natural killer cells are most commonly used in clinical trials. However, the frequency of natural killer cells available in peripheral and cord blood is low, necessitating development of methods for expansion of natural killer cells for clinical use. The development of a platform for the expansion of large-scale good manufacturing practice-compliant natural killer cells has limitations as several methods for natural killer cell activation and expansion yield conflicting results. Only techniques using feeder cells can produce large numbers of cells, allowing the “off-the-shelf” use of natural killer cells. However, advances in cell culture have supported the development of feeder-free platforms for natural killer cell expansion, which is fundamental for improving the safety of this type of cell therapy.

## INTRODUCTION

Natural Killer (NK) cells are innate lymphocytes characterized by rapid immune responses against tumors and virus-infected cells. The cytotoxicity of NK cells is mediated by different mechanisms, such as the release of cytotoxic granules (granzyme and perforin), activation of apoptotic pathways (such as TRAIL and Fas/FasL), antibody-dependent cell-mediated cytotoxicity (ADCC) via CD16 receptor, and cytokine production (mainly IFN-γ and TNF-α).^(1)^

The main sources of NK cells are peripheral blood mononuclear cells (PBMCs) and umbilical cord blood (UCB). Natural Killer cells represent 10% of the PBMC leukocytes and up to 30% of the UCB leukocytes.^(1,2)^ Alternatively, NK cells can be generated *in vitro* from induced pluripotent stem cells (iPSCs).^(3)^Finally, several NK cell lines have been reported, including HANK-1, KHYG-1, NK-92, NK-YS, NKL, SNK-6, and YT. Natural Killer-92 is the only FDA-approved cell line for clinical trials; however, these cells require irradiation before being infused into patients.^(4,5)^

Natural Killer cells have been emerging as promising tools for cancer therapy due to their intrinsic cytotoxicity against tumor cells and the availability of different sources that show reduced development of graft *versus* host disease (GVHD). Clinical outcomes have shown increased disease free survival (DFS) rates in patients with different types of cancers treated with NK cells.^(6,7)^

However, despite promising clinical results, optimization of large-scale good manufacturing practice compliant (GMP)-compliant NK cell expansion methods has some limitations. Several techniques for NK cell activation and expansion have been described in previous studies. Some studies reveal a significant potential for NK cell expansion, whereas others demonstrate low rates of cell proliferation. The variability of donors and culture conditions (*e.g*., media, cytokines, and stimulation with feeder cells) may be associated with these differences.

In this article, we review the major clinical trials that used NK cells for cancer treatment. We also analyze the global distribution of this cell therapy. Additionally, we discuss the main mechanisms of NK cell activation and expansion and focus on the advantages and disadvantages of each mechanism for clinical applications. Our analyses were based on search results obtained from the ClinicalTrials.gov platform on July 6, 2022. The following search queries were used: (a) condition or disease, Leukemia OR tumor OR cancer OR myelodysplastic OR Hematology OR Hematologic OR glioblastoma AND (b) Intervention/treatment: NK OR natural killer.

(https://clinicaltrials.gov/ct2/results?cond= Leukemia+ OR+tumor+OR+cancer+OR+myelodysplastic+ OR+Hematology+OR+Hematologic+OR+ gioblastoma&term=&intr=NK+OR+natural+killer& cntry=&state=&city=&dist=&Search=Search).

### Landscape of clinical use of natural killer cells for cancer treatment

**Figure 1:**
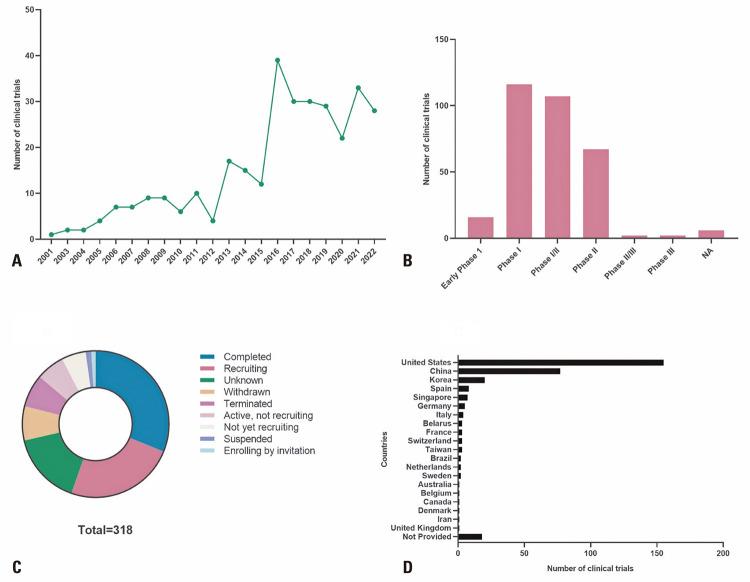
Clinical trials using natural killer cells for cancer treatment. (A) Number of clinical trials over the years; (B) Phases and (C) Status of clinical trials using natural killer cells for cancer treatment; (D) Countries using natural killer cells to treat cancer

Clinical studies on NK cells in cancer treatment have been conducted since the early 2000s (Figure 1A) with an increase in trial numbers since 2015 (Figure 1A), due to improved cell culture methods. Several clinical trials are currently in phases I and II, as this is a recent field of study, most of these studies have been completed or are in the process of recruiting patients (Figures 1B and C). Despite being a promising therapeutic tool, the use of NK cells to treat patients with cancer remains limited to developed countries, and the major countries using this technology are the United States, China, and South Korea (Figure 1D).

**Figure 2:**
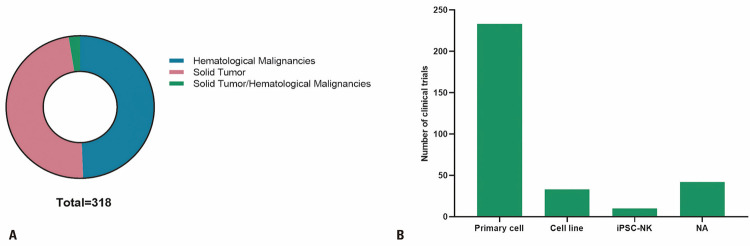
Characteristics of tumors and natural killer cells used in clinical trials. (A) Types of cancers treated using natural killer cells and (B) sources of natural killer cells

Interestingly, the number of clinical studies using NK cells to treat patients with solid tumors was similar to the number of studies on patients with hematological diseases treated with these cells (Figure 2A). Several types of solid tumors are treated using NK cells, such as glioblastomas, cervical cancer, lung cancer, and ovarian cancer. Among hematological disorders, acute myeloid leukemia (AML) is most frequently treated using NK cells (Table 1S, Supplementary Material).

The main sources of NK cells used in clinical trials were primary cells isolated from PBMCs or UCB, followed by those isolated from various cell lines (Figure 2B). Few studies used iPSC-derived NK cells (n=10) (Figure 2B). In recent decades, UCB has emerged as a promising source of NK cells for clinical use. Along with a higher percentage than that obtained from other sources, NK cells obtained from UCB revealed a greater expansion potential than that of NK Cells obtained from PBMCs. Reina-Ortiz et al.^(8)^ demonstrated that while NK cells derived from PBMCs showed 200-fold expansion, those produced using UCB attained 700-fold expansion after 20 day in culture. Herrera et al.^(9)^ observed a moderately higher fold expansion in NK cells obtained from UCB than that in NK cells obtained from PBMCs after 2 weeks of *in vitro* expansion. Furthermore, thousands of cryopreserved UCB units are readily accessible in several cell banks worldwide, which can be used as an alternative to freshly isolated PBMCs or UCB as a source of NK cells.^(10)^

### Current methods for the activation and expansion of natural killer cells

**Figure 3:**
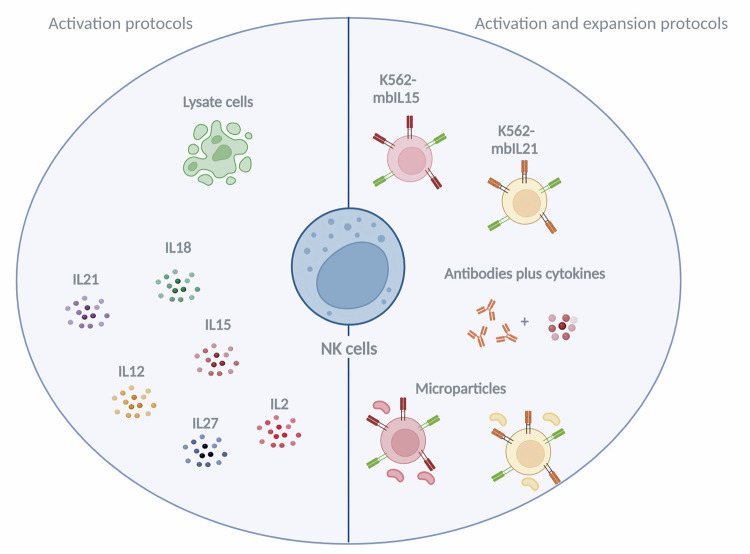
Methods for the activation or activation and expansion of natural killer cells. Several techniques have been developed only for the activation of natural killer cells and IL-2, IL-12, IL-15, IL-18, IL-21, and IL-27 are the cytokines used commonly. Other methods focusing on natural killer cell expansion include the use of antibodies and feeders together with cytokines

Development of methods for expansion of large-scale GMP-compliant NK cells as a prospective therapy for cancer has limitations. Several methods have been developed to activate and expand NK cells and have yielded varying results. As primary cells are the most common type of NK cells used in clinical trials, we focused on reviewing methods for the activation and expansion of these cells (Figure 3). NK cells are isolated and activated using different cytokines (individually or in combination) for a few hours (Figure 3). Commonly used cytokines for NK cell activation are IL-2, IL-12, IL-15, IL-18, IL-21, and IL-27.^(6,11,12)^ Despite no increase in the number of NK cells or no yield of batches of cells for several infusions, this activation method has shown promising results.

Created with the tool BioRender. Available from: https://www.biorender.com/

Lee et al.^(6)^ treated myeloid malignancies with third-party haploidentical NK cells activated overnight with IL-2 prior to allogeneic hematopoietic stem cell transplantation (HSCT).^(6)^ This study proved that the infusion of NK cells was safe and did not affect engraftment or GVHD rate. In addition, increased relapse-free survival was associated with increased NK cell doses.^(6)^

In addition to IL-2, other cytokines have been shown to be important for the activation and expansion of NK cells. *In vitro* studies revealed that treatment using IL-15/IL-18/IL-27 improved NK cell expansion and resulted in NK cells with increased cytotoxicity and IFN-γ production, indicating the potential of these cytokines in NK cell activation and expansion.^(11)^ Romee et al.^(12)^ demonstrated that *in vitro* pre-activation of peripheral NK cells for 16 h with IL-12, IL-15, and IL-18 resulted in the induction of cytokine-induced memory-like (CIML) NK cells, which showed increased production of IFN-γ and cytotoxic activity against tumor cells *in vitro* and *in vivo* compared to those of NK cells without activation using IL-12, IL-15, and IL-18.^(12)^ Furthermore, six patients with AML who had relapsed after allogeneic HSCT were preconditioned with fludarabine/cyclophosphamide and treated with different doses of HLA-haploidentical CIML NK cells, followed by administration of several low doses of IL-2 every alternate day. Natural killer cell numbers expanded by a mean of 10-fold and the increased number of NK cells persisted for up to 2 months after infusion.^(13)^

Recently, the use of the leukemia cell line CTV-1 lysate has emerged as an alternative for cytokines for short-term activation of NK cells *in vitro.*^(7)^ In a phase 1 clinical trial, patients with high-risk AML in first complete remission were treated with fludarabine/cyclophosphamide and cryopreserved HLA-haploidentical NK cells activated for 16 h with a CTV-1 cell lysate (CNDO-109). This study revealed that treatment with cryopreserved CNDO-109-NK cells was safe and that NK cells persisted transiently, resulting in long-term relapse-free survival.^(7)^

In contrast, methods developed for NK cell expansion allow the recovery of a large number of activated NK cells, which can be used immediately upon isolation or cryopreserved samples can be used in several infusions for different types of tumors, thereby increasing the use of “off-the-shelf” NK cells in cancer immunotherapy. Several NK cell expansion methods have been developed in recent years, and the potential for NK cell expansion depends on the type of activator (beads, antibodies, or feeder cells) and cytokine (IL-21 and IL-15) (Figure 3).

Some studies have shown that antibodies can stimulate *in vitro expansion of NK cells*. Masuyama et al.^(14)^ used anti-CD3 and anti-CD52 mAbs and IL-2 to activate PBMCs from healthy donors. Natural killer cell expansion was observed to be 646-fold on day 14 of culture, and the cytotoxic activity of the expanded NK cells increased *in vitro* and in an animal model of human pancreatic cancer. One patient with pancreatic cancer who was administered multiple infusions of autologous NK cells exhibited an increased survival rate, although he died 13 months after NK cell infusion, suggesting the importance of several doses of NK cells over a long duration to prolong overall survival.^(14)^ Recently, six patients with multiple myelomas underwent autologous HSCT and were subsequently administered multiple doses of NK-activated cells which had been expanded using anti-CD3 and IL-2.^(15)^

Another way to increase the number of NK cells is to use feeder cells. Feeder cells are growth-arrested cells that support the growth of cells of interest through cell contact or release of growth factors.^(16)^ In general, irradiation and mitomycin-C treatment are the most commonly used methods to suppress feeder cell proliferation. Natural killer cell expansion using feeder cells results in a higher fold-increase and is the most common platform used in clinical trials (Table 1S, Supplementary Material). The irradiated K562 cell line was used to expand the NK cells. These cells were genetically engineered to express activation molecules (such as 4-1BBL) and membrane-bound cytokines (such as mbIL-15 or mbIL-21) and were irradiated (to avoid their growth) before co-culture with NK cells.

Many studies have used K562-mb15-41BBL feeder cells to expand NK cells and have tested the cytotoxic capacity of NK cells *in vitro* and *in vivo*. Peripheral blood mononuclear cells isolated from healthy donors and patients with solid tumors were expanded with irradiated K562-mbIL15-41BBL feeder cells after 14 days. Natural killer cells from healthy donors expanded 165-fold on an average and the percentage of NK cells was 45.6%, whereas the percentage of T cells was 30.0% at the end of the culture. Natural killer cells from patients expanded approximately 316-fold, and the percentage of NK cells was 80% on day 14. In addition, the expanded NK cells were highly cytotoxic against prostate and breast cancer cell lines *in vitro.*^(17)^ Peripheral blood mononuclear cells from healthy donors and patients with AML were cultured with irradiated K562-mbIL15-41BB.^(18)^ Natural killer cells from healthy donors expanded an average of 21.6-fold by day 7, increased to 152-fold by day 14, and 277-fold after 21 day of culture, whereas the percentages of NK cells were 62.9%, 90.0%, and 96.8%, respectively. Similarly, the fold-expansion of NK cells from patients was 17.3-fold on day 7 of culture.^(18)^ Furthermore, expanded NK cells showed a high cytotoxic response against tumor cells *in vitro* and *in vivo* and persisted *in vivo* after IL-2 stimulation.^(18)^

In addition to the *in vitro* and *in vivo* tests, clinical trials using NK cells expanded using K562-mb15-41BBL feeder cells were conducted (Table 1S, Supplementary Material). Patients with relapsed or refractory childhood leukemia/lymphoma were treated with lymphodepletion chemotherapy, followed by two or four infusions of NK cells from haploidentical donors. Of the 20 patients recruited, 15 were alive at the end of follow-up, of which six showed complete remission (CR/MRD-), seven presented complete remission (CR/MRD+), and two did not respond to therapy.^(19)^Next, in a phase II clinical trial, seven patients with low-to intermediate-risk AML in the first complete remission were treated with two infusions of activated NK cells isolated from haploidentical donors.^(20)^ The median fold increase was observed to be 56.97 after 3 weeks of expansion *in vitro*. Of the seven patients treated with NK cells, six remained alive with their disease in complete remission thousand days after treatment with NK cells, and the overall survival was 83.3% 3 years after NK cell therapy.^(20)^

In addition to allogeneic NK cells, autologous NK cells were expanded using K562-mb15-41BBL feeder cells used in combination with lenalidomide or bortezomib to treat patients with multiple myelomas.^(21)^ Autologous NK cells expanded 45-fold (mean) with a purity of 90% after 21 day in culture and were highly cytotoxic *in vitro.* Three patients exhibited stable disease and one had a partial response after treatment with several doses of autologous NK cells (in combination with anti-myeloma drugs). One patient was excluded because of an unrelated complication.^(21)^ Autologous NK cells in combination with cetuximab have been used to treat patients with solid tumors. Peripheral blood mononuclear cells from patients with nasopharyngeal carcinoma were cultured with K562-mb15-41BBL feeder cells, and NK cells expanded 200-fold in 10 days and showed high expression of CD16 (98.4%), which is important for improving the mechanism of action of cetuximab. Three patients showed disease progression and four exhibited stable disease after treatment. Disease progression was relatively slower in patients who received two doses of NK cells than that seen in patients who received one dose.^(22)^ Patients with HER2+ metastatic breast cancer were also treated with expanded NK cells and trastuzumab. Seven of the ten patients had stable disease, and analysis of tumor biopsies revealed lymphocyte infiltration after NK cell infusion.^(23,24)^

In addition, clinical studies on NK cells expanded using K562-mb21-41BBL feeder cells have revealed promising results. K562-mb21-41BBL feeder cells improved NK cell expansion and prevented telomere shortening and senescence.^(25)^ Furthermore, NK cells expanded using K562-mb21-41BBL feeder cells showed a mean 47,967-fold expansion, whereas NK cells expanded using K562-mb15-41BBL feeder cells exhibited a mean 825-fold expansion. Finally, the phenotype and function of NK cells expanded using mbIL21 were similar to those expanded using mbIL15, resulting in elevated expression of natural cytotoxicity receptors (NCRs), CD16, and NKG2D, as well as in increased cytokine secretion.^(25)^

The safety and efficacy of high-dose mbIL21 *ex vivo* expanded donor-derived NK cells were investigated in a phase 1 clinical trial, including patients with myeloid malignancies receiving NK cell infusion after haploidentical HSCT.^(26)^ Of the 11 patients participating, seven developed grade 1-2 acute GVHD, none developed grade 3-4 acute GVHD or chronic GVHD, and a low incidence of viral complications was observed. One patient died of no relapse mortality, one patient relapsed, and all others were in remission at the time of the last follow-up (median 14.7 months). Natural killer cell reconstitution was quantitatively, phenotypically, and functionally superior compared to that seen in a similar group of patients not receiving NK cells.^(26)^ In a phase I/II clinical trial, including 25 patients, the 2-year relapse rate was 4% *versus* 38% (p=0.014), and the DFS was 66% *versus* 44% (p=0.1) in patients treated with NK cells and controls, respectively. NK cells in recipient blood presented a mature and highly cytotoxic phenotype, which increased on day 30 in a dose-dependent manner compared to that of controls.^(27)^ Interestingly, patients with relapsed or refractory AML and concurrent central nervous system diseases treated with haploidentical NK cells (mbIL21 expanded) exhibited long-lasting clinical outcomes.^(28)^ Patients with multiple myelomas treated with autologous HSCT plus expanded cord blood derived NK cells also exhibited improved outcomes, with 55% patients achieving a complete response, and 12% and 21% showing notable partial response 3 months after treatment, respectively.^(29,30)^

In addition to studies using NK-expanded cells to treat myeloid malignancies, a phase I study was performed using intraventricular infusions of autologous *ex vivo* expanded NK cells in 12 children with recurrent medulloblastoma and ependymoma.^(31)^ Nine patients received three infusions weekly, and all patients showed progressive disease, except one patient who showed stable disease for 1 month at the end of the study upon follow-up. Interestingly, at high doses, NK cells induced an increase in volumes of cerebrospinal fluid during treatment with repetitive infusions (mean 11.6-fold) and frequent infusions of NK cells resulted in cerebrospinal fluid pleocytosis.^(31)^

Recently, Oyer et al. proposed a technology that uses plasma membrane particles (PM particles) derived from K562-mbIL15-41BBL or K562-mbIL21-41BBL feeder cells for NK cell expansion. The benefit of this platform is that it is a feeder-free system in which PM can be prepared consistently and stored at -80°C for at least 1 year. First, we wanted to compare PM particles derived upon K562-mbIL15-41BBL cells (PM15) for expansion of NK cells with their expansion from those obtained using conventional expansion methods. Peripheral blood mononuclear cells were cultured with PM15 or K562-mbIL15-41BBL cells. The average NK cell expansion was 572-fold on day 16 upon using PM15 and 396-fold when they were cultured with feeder cells. In addition, stimulation with PM15 was specific to NK cells, while T and B cell counts decreased during culture, and PM15-NK cells were as cytotoxic as NK cells obtained using feeder cells.^(32)^ Next, we studied the potential of PM particles derived upon using K562-mbIL21-41BBL (PM21) to activate and expand NK cells, as IL-21 has been associated with enhanced expansion of NK cells without senescence induction.^(30)^ Natural killer cells activated using PM21 increased 825-fold compared to a mean of 424-fold increase when PM15 particles were used. Furthermore, NK cells stimulated with PM21 particles expanded exponentially in 28 d of culture, whereas NK cell expansion upon use of PM15 particles reached a plateau by day 22 of culture owing to senescence.^(30)^Lastly, studies with animal models demonstrated that the infusion of PBMCs activated with PM21 resulted in NK cell expansion *in vivo* and that the treatment of animals with PM21 induced NK cell expansion, suggesting the potential use of PM21 for *in vivo* expansion of NK cells.^(33)^

In addition to K562 feeder cells, the activation of peripheral blood NK cells with a clinical-grade irradiated Epstein-Barr virus-transformed lymphoblastoid cell line (EBV-LCL), with IL-2 and IL-21 resulted in a 53-fold expansion after one week of culture.^(31)^Interestingly, the addition of IL-2 and IL-21 at the beginning of the culture, followed by repeated stimulation with irradiated EBV-LCL feeder cells every 2 weeks, resulted in a 2.7 × 10^11^-fold increase in NK cell counts after 46 days. NK cells expanded using EBV-LCL feeder cells (with IL-2 and IL-21) showed increased cytotoxic rates *in vitro* as well as significant antitumor activity and persistence in melanoma animal models.^(34)^

Moreover, Ojo et al.^(35)^created a new feeder cell platform (NKF) for NK cell expansion, comparable to the K562 platform.^(35)^ NKF cells are OCI-AML3 (a myeloid leukemia cell line) expressing IL-21 (mbIL-21), and these cells were able to induce a huge expansion of NK cells (over 10,000-fold) after 5 weeks of culture. Natural killer cells expanded using NKF showed an increased *in vitro* cytotoxic response against diverse types of hematological and solid tumor cells and great efficacy in animal models of sarcomas and T-cell leukemia.^(35)^ Natural killer cells expanded using NKF were used to treat nine patients (three with MDS or AML and six with colorectal carcinoma). Patients were prepared using a lymphocyte-depleting chemotherapy regimen, followed by NK infusion. A second dose of NK cells was administered 2 weeks later, and the patients were monitored for up to 100 days after NK cell treatment. The administration of NK cells was safe (no GVHD or other toxicities were observed), and one patient had a complete response, while three other patients exhibited stable disease, demonstrating the potential of NK cells for cancer treatment.^(36)^

Finally, some research groups have proposed the use of PBMCs as feeder cells to avoid the use of cell lines and increase the safety of cell products. Torelli et al.^(37)^ proposed a method consisting of peripheral blood NK cells cultured with irradiated autologous feeders (monocytes, T cells, and B cells) for 14 days in a medium containing autologous plasma; IL-2, IL-15, and NK cells expanded on an average 15.7-fold. After expansion, NK cells showed significant upregulation of activating receptors and KIR inhibitors as well as an increased cytotoxic response against the K-562 cell line (fresh and cryopreserved NK cells).^(37)^ In another study, peripheral blood NK cells were cultured with irradiated autologous PBMCs for 14 day; however, instead of IL-15, the authors used IL-2 plus anti-CD3 (defined as MG4101). Natural killer cell expansion observed was 757.5-fold and these cells exhibited increased cytolytic activity against K562 cells. In addition, MG4101 toxicity was evaluated in a clinical trial involving patients with malignant lymphomas or advanced solid tumors. After NK infusion, eight patients exhibited stable disease and nine patients had progressive disease; however, no toxicity related to MG4101 administration was observed.^(38)^

Recently, an autologous NK cell expansion system was developed using recombinant human fibronectin fragment (FN-CH296)- induced T cells (RN-T cells) as stimulators.^(39)^ Autologous PBMCs were isolated and introduced to a cell culture immobilized with anti-CD3 mAb and FN-CH296 for 1-2 weeks, when the cells were γ-irradiated. Next, RN-T cells were cultured with autologous PBMCs for 21 or 22 d. At the end of the culture period, NK cells accounted for more than 90% of the cells, and the median cell expansion was 586-fold and 4720-fold on days 21 and 22 of culture, respectively. Patients with advanced digestive cancers treated with NK cells expanded using RN-T cells did not experience any severe or unexpected toxicity, and the cytotoxicity of PBMCs against K-562 cells increased by 80% after the transfer of adoptive NK cells. However, none of the patients showed a decrease in tumor size 4 weeks after the last infusion.^(39)^ Based on this, Ishikawa et al.^(40)^ evaluated the safety, toxicity, and immunological responses to autologous NK cells expanded using RN-T cells in combination with trastuzumab or cetuximab in patients with advanced gastric or colorectal cancers.^(40)^ After treatment, 50% patients showed a clinical response, such as disease stabilization and a decrease in lesion size and serum CEA levels.^(40)^

## CONCLUSION AND PERSPECTIVES

Development of methods for expansion of large-scale good manufacturing practice-compliant natural killer cells is promising as a therapy for various cancers. Several methods have been developed to activate and expand natural killer cells. *Ex vivo* natural killer cells are used for immunotherapy because of their high anticancer activity and low adverse effects and toxicity. The number of clinical trials using these cells has increased in recent years and both, patients with hematological diseases and solid tumors have been aided, although the treatment of solid tumors has some limitations. Natural killer cell infusion was shown to be safe and did not affect engraftment or graft *versus* host disease rate. Immunotherapy using natural killer cells in patients with solid tumors remains a major challenge owing to the limited availability of tumor-specific antigens and the immunosuppressive state of the tumor microenvironment. A combination of natural killer cells with other immunotherapies, such as monoclonal antibodies, immunomodulatory drugs, and cytokines, may enhance the natural killer-mediated anti-tumor response.^(41,42)^

Although various methods of activation and expansion of primary natural killer cells are now available, only the use of feeder cells aids production of large numbers of cells that can be used immediately upon isolation as well as cryopreserved for subsequent therapy, allowing “off-the-shelf” use of natural killer cells. However, advances in cell culture have supported the development of feeder-free platforms, such as plasma membrane particles derived from feeder cells for natural killer cell expansion, which will result in significant improvements in the safety of this type of cell therapy.

Finally, despite *ex vivo* natural killer cell expansion resulting in the development of highly cytotoxic natural killer populations, *ex vivo* expansion and genetic engineering of natural killer cells (such as chimeric antigen receptor-natural killer cells) improve their activation and cytotoxic functions. Chimeric antigen receptor-natural killer therapies have demonstrated some advantages, such as a lower risk of graft-*versus*-host disease, reduced side effects, “off-the-shelf” availability, and intrinsic antibody-dependent cell-mediated cytotoxicity.^(42)^ Thus, advances in natural killer-based immunotherapies are considered promising for cell therapy, especially in patients with hematological cancers. Additionally, the use of natural killer cells combined with other therapies and/or with cutting-edge genetic engineering strategies, such as chimeric antigen receptor non-cleavable CD16 Fc receptors, and silencing TGF-β receptors, will be useful for cancer treatment.
